# Immunotherapy and gene therapy in prostate cancer treatment

**Published:** 2017

**Authors:** T Grozescu, F Popa

**Affiliations:** * “Carol Davila” University of Medicine and Pharmacy, Bucharest, Romania

**Keywords:** immunotherapy, prostate cancer treatment, lifespan, neoplastic tissue, gene therapy

## Abstract

There are few methods bringing several relatively recent advances in therapy of certain types of prostate cancer. Belonging to personalized therapies, they use cells (normal or pathologic) from the patient, modify and reintroduce them in the patient’s body, leading to an increased efficiency against the neoplastic tissue, proving to increase the patient’s lifespan and/ or tumor progression.

The only drug to have the FDA approval for clinical use (February 20, 2015) until now is Sipuleucel-T (Provenge) [**1**-**4**], a therapeutic vaccine manufactured through ex vivo gene therapy. It addresses advanced hormone-refractory prostate cancer patients (metastasized) (mHRPC), showing an increase of the lifespan (versus placebo) of approximately 4 months (on average from 21 to 25 months) in all three phases of clinical testing. Both the principle and the course of treatment consist of three steps [**5**,**6**]:

• Extracting a population of lymphocytes, mainly dendritic cells (this type of cells are antigen-presenting cells, APCs), from the patient;

• The isolate, basically the dendritic cells, is incubated with a fusion protein, named PA2024, consisting of a PAP region (the antigen of prostatic acid phosphatase, found in more than 95% of the prostate cancer cells) and a stimulating region (granulocyte-macrophage colony stimulating factor GM-CSF), which helps the dendritic cells (APCs) mature;

• The product, APC8015, is infused to the patient within three sessions at two weeks intervals.

Interestingly, Sipuleucel-T (Provenge) is the first therapeutic vaccine generally approved for the treatment of any type of cancer [**7**-**10**].

Other immunotherapies, still undergoing different stages of clinical testing, include a large number of vaccines, administered alone or in combination with other therapies. From the most advanced, the following are mentioned:

• Prostvac (rilimogene galvacirepvec) is also a therapeutic vaccine, found in phase III of a large randomized clinical trial. It uses a virus (vaccinia) as a vector to deliver the prostate specific antigen (PSA) altogether with other three co-stimulating molecules (LFA-3, ICAM-1 and B7.1) directly to cancer cells. The viral vector stimulates an immune response against PSA, directing the immune system to attack the prostate cancer cells. Its addressability is similar to Provenge, namely the metastatic hormone-resistant prostate cancers (mHRPC); yet showing promising results within phase II clinical trials, with an increase of the median overall survival of 8.5 months, double compared to Provenge. Phase III testing started in November 2011 and results are expected by the end of this year [**4**-**7**].

• DCVAC/ PCa, also found in phase III of clinical testing, is a vaccine containing autologous dendritic cells activated by ex vivo exposure to killed LNCaP cells (one of the three most known cell lines of prostate cancer). It has been tested in an extended randomized clinical trial started in May 2014, enrolling more than 1200 patients in approximately 230 locations worldwide. The study was projected to seek the efficiency of the vaccine administered together with chemotherapy (Docetaxel/XT/Taxotere/Docecad) in patients with metastatic hormone-resistant prostate cancers, as well (mHRPC) [**5**-**9**].

• Most of the efforts target advanced prostate cancer which has few other therapy options, otherwise localized cancers are either indolent and undergo active surveillance, or benefit from a surgical (combined with chemo or radiotherapy) treatment, plus the ones with the hormone replacement therapy option.

**Oncolytic virus therapy**

By definition, it uses genetically modified viruses that can induce destruction of infected neoplastic cells, in this process generating also an immune antitumor response, greater than other therapies.

• ProstAtak (aglatimagene besadenovec) uses an inactivated herpetic virus to directly deliver a gene to cancer cells, followed by an anti-herpetic therapy with valacyclovir (Valtrex), killing the cells containing the gene. Currently, ProstAtak is undergoing phase III of clinical testing in patients with localized prostate cancer who also follow radiotherapy. There is also a phase II/ III clinical trial using ProstAtak in patients with localized prostate cancer following only active surveillance (conservative management). Both studies have been started in September 2011 and are expected to be finalized in December 2019 [**7**,**9**]. 

**Adoptive Cell Therapy**

 Another important branch of immunotherapy in prostate cancer is adoptive cell therapy. This involves the extraction of immune cells from the patient, modifying them genetically, or treating them with substances meant to enhance their activity, then reinserting them in the patient’s body, with the purpose of obtaining a better antitumor response [**5**,**6**].

• A phase II clinical trial uses genetically modified T cells in order to target NY-ESO-1, the prostate cancer specific antigen, administered together with a vaccine containing dendritic cells activated for NY-ESO-1, for a greater efficiency [**7**,**9**].

• A phase I clinical trial uses genetically modified NK (Natural Killer) cells [**7**,**9**].

**Adjuvant immunotherapies**

Are based on drugs that boost the immune antitumor response. They can be used alone or in combination with other immunotherapies [**5**,**6**].

• A phase II clinical trial uses Sipuleucel-T (Provenge) and Indoximod simultaneously, an indoleamine (IDO) pathway inhibitor in patients with refractory metastatic prostate cancer. The indoleamine (IDO) pathway is frequently active in tumors, and Indoximod is known to inhibit this pathway [**5**-**9**].

• A phase I clinical trial uses Mobilan (M-VM3), a toll-like receptor agonist [**7**,**9**].

**Monoclonal antibodies**

Monoclonal antibodies are molecules designed and made in the lab by genetic engineering, meant to target specific tumor antigens. Currently, a large number of monoclonal antibodies are undergoing clinical trials, some with promising results; yet these studies are still in the preliminary phases [**5**,**6**,**9**].
